# Frequent neurologic manifestations and encephalopathy‐associated morbidity in Covid‐19 patients

**DOI:** 10.1002/acn3.51210

**Published:** 2020-10-05

**Authors:** Eric M. Liotta, Ayush Batra, Jeffrey R. Clark, Nathan A. Shlobin, Steven C. Hoffman, Zachary S. Orban, Igor J. Koralnik

**Affiliations:** ^1^ Ken & Ruth Davee Department of Neurology Northwestern University Feinberg School of Medicine Chicago Illinois USA

## Abstract

**Objective:**

Covid‐19 can involve multiple organs including the nervous system. We sought to characterize the neurologic manifestations, their risk factors, and associated outcomes in hospitalized patients with Covid‐19.

**Methods:**

We examined neurologic manifestations in 509 consecutive patients admitted with confirmed Covid‐19 within a hospital network in Chicago, Illinois. We compared the severity of Covid‐19 and outcomes in patients with and without neurologic manifestations. We also identified independent predictors of any neurologic manifestations, encephalopathy, and functional outcome using binary logistic regression.

**Results:**

Neurologic manifestations were present at Covid‐19 onset in 215 (42.2%), at hospitalization in 319 (62.7%), and at any time during the disease course in 419 patients (82.3%). The most frequent neurologic manifestations were myalgias (44.8%), headaches (37.7%), encephalopathy (31.8%), dizziness (29.7%), dysgeusia (15.9%), and anosmia (11.4%). Strokes, movement disorders, motor and sensory deficits, ataxia, and seizures were uncommon (0.2 to 1.4% of patients each). Severe respiratory disease requiring mechanical ventilation occurred in 134 patients (26.3%). Independent risk factors for developing any neurologic manifestation were severe Covid‐19 (OR 4.02; 95% CI 2.04–8.89; *P* < 0.001) and younger age (OR 0.982; 95% CI 0.968–0.996; *P* = 0.014). Of all patients, 362 (71.1%) had a favorable functional outcome at discharge (modified Rankin Scale 0–2). However, encephalopathy was independently associated with worse functional outcome (OR 0.22; 95% CI 0.11–0.42; *P* < 0.001) and higher mortality within 30 days of hospitalization (35 [21.7%] vs. 11 [3.2%] patients; *P* < 0.001).

**Interpretation:**

Neurologic manifestations occur in most hospitalized Covid‐19 patients. Encephalopathy was associated with increased morbidity and mortality, independent of respiratory disease severity.

## Introduction

As of 8 September 2020, severe acute respiratory syndrome coronavirus type 2 (SARS‐CoV‐2) has led to over 26.5 million confirmed infections and 875,000 deaths from coronavirus disease‐2019 (Covid‐19) worldwide.^1^

Like most infections caused by members of the coronavirus family, SARS‐CoV‐2 manifests itself with upper respiratory tract infections and flu‐like symptoms of varying severity.^2^ However, Covid‐19 is unique in its ability to cause a multi‐organ disease, with involvement of the central and peripheral nervous system in some individuals.

Indeed, a wide range of neurologic manifestations of SARS‐CoV‐2 infection have been recognized, and evidence of their severity and persistence is increasing.^3^, ^4^, ^5^, ^6^, ^7^, ^8^, ^9^, ^10^ However, the frequency of those manifestations and associated risk factors remain unclear. We sought to characterize the incidence of neurologic manifestations, in patients with confirmed Covid‐19 and identify factors associated with the development of neurologic manifestations in hospitalized patients with both severe and non‐severe respiratory disease. Furthermore, neurologic manifestations, especially encephalopathy, have been associated with worse clinical outcomes in other systemic illnesses including sepsis and may even lead to significant disability.^11^, ^12^ Therefore, we sought to identify if encephalopathy was associated with greater morbidity in hospitalized patients with Covid‐19.

## Subjects/Materials and Methods

### Patients

We retrospectively analyzed the first consecutive patients admitted with Covid‐19 to the Northwestern Medicine Healthcare (NMHC) system between 5 March and 6 April 2020. NMHC consists of one academic medical center (AMC) and nine other hospitals in the Chicago area. Covid‐19 diagnosis was confirmed by SARS‐CoV‐2 reverse transcription‐polymerase chain reaction (RT‐PCR) assay of nasopharyngeal swab or broncho‐alveolar lavage fluid. All laboratory and radiologic assessments were performed as part of routine clinical care. This study was approved by our institutional review board (STU00212627) with waiver of consent for retrospective analysis.

### Procedures

Demographic, medical comorbidity, pre‐hospitalization medication usage, and hospital course data were collected by electronic medical record review. Laboratory data were collected by automated electronic query. Neurologic manifestations were identified by review of clinical notes, diagnostic studies, and physician‐documented diagnoses. The identification of neurologic manifestations, the dates of neurologic manifestation onset, and Covid‐19 symptom onset dates was facilitated by electronic note templates implemented in the NMHC System as part of the Covid‐19 response. In particular, encephalopathy was identified by (a) report of altered mental status or depressed level of consciousness, (b) physician documented diagnosis of encephalopathy or the delirium encephalopathy syndrome, or (c) positive Confusion Assessment Method (CAM) evaluation. The Confusion Assessment Method is a well‐validated and widely used clinical and research tool for the identification of the delirium encephalopathy syndrome and has been in routine clinical use at NMHC since 2008.^13^, ^14^, ^15^ Neurologic manifestations were included starting on the date of Covid‐19 onset, as identified by the patient’s clinical provider, through 90 days. In cases where neurologic manifestations were not specifically attributed to a documented diagnosis, the cases were independently adjudicated by two separate neurologists (AB and EML). A third neurologist independently reviewed the chart to serve as a tie‐breaker (IJK) in cases of disagreement. In those cases where neurologic manifestations were attributed to a specific neurologic diagnosis (e.g., stroke), the patient was recorded as having that specific diagnosis rather than each component neurologic manifestation. Patients were dichotomized into severe versus non‐severe Covid‐19 respiratory disease based on the need for mechanical ventilation during hospitalization; this criterion was used to account for the possibility of delayed clinical deterioration after hospital admission.

Functional outcome at hospital discharge was extracted from therapy and rehabilitation medicine physician documentation using the modified Rankin Scale (mRS) categorized as: mRS 0–2, able to look after ones’ own affairs without assistance; mRS 3, ambulates unassisted but needs help with ones’ own affairs; mRS 4–5, unable to ambulate unassisted and needs assistance for bodily care; and mRS 6, death. The discharge mRS scores were determined independently by two reviewers and disagreements were reconciled by majority decision after review (EML).

### Statistical analysis

Data were summarized as number of patients (frequency), mean (standard deviation) for normally distributed variables, and median (interquartile range [IQR]) for non‐normally distributed variables. Associations were assessed using Fisher’s exact test, Spearman’s rank correlation test, and Wilcoxon rank‐sum test. Binary logistic regression models were developed to identify adjusted predictors of: (1) experiencing any neurologic manifestations, (2) experiencing encephalopathy, and (3) having a favorable discharge functional outcome (mRS 0–2). In each case, we first developed a model using *a priori* variables based on biologic plausibility and review of the recent Covid‐19 literature. Then we developed models using the *a priori* variables plus additional variables univariately associated with the model outcome at *P* ≤ 0.15 and not highly collinear with a variable already included. To avoid overfitting models to the data, we used a backward variable selection algorithm based on Akaike Information Criteria optimization to develop final *parsimoniously adjusted* models. Details of the *a priori* and *parsimonious* models, including the *a priori* variables for each model, are included in the Table S2. We also developed an *a priori* binary logistic regression model for the association between encephalopathy and 30‐day mortality adjusted for age, severe Covid‐19 disease, and hospitalization at the AMC; limited numbers of patient deaths prevented inclusion of additional variables for adjustment. Two‐sided *P* ≤ 0.05 was considered significant and all analyses were performed in R version 3.5.0 (R Foundation for Statistical Computing, Vienna, Austria).

## Results

### Frequency of neurologic manifestations

There were 509 consecutive patients included in the study (age 58.5 ± 16.9 years, 281 (55.2%) males), and 134 (26.3%) had severe Covid‐19 requiring mechanical ventilation. Neurologic manifestations were present at Covid‐19 onset in 215 patients (42.2%), at hospital admission in 319 patients (62.7%) and at any time during the disease course in 419 patients (82.3%). The most frequent neurologic manifestations were myalgias (228, 44.8%), headaches (192, 37.7%), encephalopathy (162, 31.8%), dizziness (151, 29.7%), dysgeusia (81, 15.9%), and anosmia (58, 11.4%). In addition, reports of generalized fatigue (214 [42.9%] patients) at onset and any time during Covid‐19 disease (404 [79.4%] patients) were common. The clinical characteristics of the patients with and without any neurologic manifestations or encephalopathy are summarized in Table 1. Patients presenting with any neurologic manifestations were younger than those without (57.53 [16.31] vs. 62.98 [18.97] years; *P* = 0.005) and had a longer time from COVID onset to hospitalization (7 [4, 10] vs. 5 [2, 9] days; *P* = 0.003). Conversely, patients with encephalopathy were older than those without (65.51 [16.54] vs. 55.22 [16.10] years; *P* < 0.001), had a shorter time from COVID onset to hospitalization (6 [3, 9] vs. 7 [4, 10] days; *P* = 0.014), were more likely to be male, and to have a history of any neurological disorder, cancer, cerebrovascular disease, chronic kidney disease, diabetes, dyslipidemia, heart failure, hypertension and smoking in assessments without multivariate adjustment. There were no differences in use of angiotensin converting enzyme (ACE) inhibitors, angiotensin 2 receptor blocker (ARB), corticosteroids or immunosuppressants prior to admission in patients with and without any neurologic manifestations or encephalopathy (Table S1).

**Table 1:** Patient characteristics by presence of neurologic manifestations and encephalopathy.

	Overall	No Neurologic Manifestation	Any Neurologic Manifestation	*P*	No Encephalopathy	Encephalopathy	*P*
*n*	509	90	419		347	162	
Age, years (mean (SD))	58.51 (16.93)	62.98 (18.97)	57.53 (16.31)	0.005	55.22 (16.10)	65.51 (16.54)	<0.001
Male, *n* (%)	281 (55.2)	50 (55.6)	231 (55.1)	1	180 (51.9)	101 (62.3)	0.034
Female, *n* (%)	228 (44.8)	40 (44.4)	188 (44.9)		167 (48.1)	61 (37.7)	
Race, *n* (%)				0.227			0.204
White	268 (52.7)	56 (62.2)	212 (50.6)		172 (49.6)	96 (59.3)	
Black or African American	151 (29.7)	26 (28.9)	125 (29.8)		114 (32.9)	37 (22.8)	
Asian	18 ( 3.5)	2 ( 2.2)	16 ( 3.8)		12 ( 3.5)	6 ( 3.7)	
Native Hawaiian or Other Pacific Islander	1 ( 0.2)	0 ( 0.0)	1 ( 0.2)		0 ( 0.0)	1 ( 0.6)	
Other	53 (10.4)	3 ( 3.3)	50 (11.9)		37 (10.7)	16 ( 9.9)	
Unknown	1 ( 0.2)	0 ( 0.0)	1 ( 0.2)		1 ( 0.3)	0 ( 0.0)	
Declined to provide	17 ( 3.3)	3 ( 3.3)	14 ( 3.3)		11 ( 3.2)	6 ( 3.7)	
Ethnicity, *n* (%)				0.075			0.23
Not Hispanic or Latino	384 (75.4)	75 (83.3)	309 (73.7)		263 (75.8)	121 (74.7)	
Hispanic or Latino	107 (21.0)	11 (12.2)	96 (22.9)		75 (21.6)	32 (19.8)	
Declined to Provide	18 ( 3.5)	4 ( 4.4)	14 ( 3.3)		9 ( 2.6)	9 ( 5.6)	
Time from COVID Onset to Hospitalization, days (median [IQR])	7.00 [4.00, 9.59]	5.00 [2.00, 8.98]	7.00 [4.00, 10.00]	0.003	7.00 [4.00, 10.00]	6.00 [3.00, 9.00]	0.014
Hospitalized at the Academic Medical Center, *n* (%)	254 (49.9)	32 (35.6)	222 (53.0)	0.004	168 (48.4)	86 (53.1)	0.375
Medical Comorbidities							
History of Any Neurological Disorder, *n* (%)	134 (26.3)	27 (30.0)	107 (25.5)	0.459	79 (22.8)	55 (34.0)	0.010
Cancer, *n* (%)	61 (12.0)	10 (11.1)	51 (12.2)	0.919	29 ( 8.4)	32 (19.8)	<0.001
Cerebrovascular Disease, *n*(%)	39 ( 7.7)	12 (13.3)	27 ( 6.4)	0.044	18 ( 5.2)	21 (13.0)	0.004
Chronic Kidney Disease, *n* (%)	56 (11.0)	10 (11.1)	46 (11.0)	1	29 ( 8.4)	27 (16.7)	0.008
Diabetes Mellitus, *n* (%)	154 (30.3)	31 (34.4)	123 (29.4)	0.408	95 (27.4)	59 (36.4)	0.049
Dyslipidemia, *n* (%)	172 (33.8)	32 (35.6)	140 (33.4)	0.789	100 (28.8)	72 (44.4)	0.001
Heart Failure, *n* (%)	36 ( 7.1)	7 ( 7.8)	29 ( 6.9)	0.951	14 ( 4.0)	22 (13.6)	<0.001
Hypertension, *n* (%)	277 (54.4)	55 (61.1)	222 (53.0)	0.198	169 (48.7)	108 (66.7)	<0.001
Organ transplantation, *n* (%)	16 ( 3.1)	2 ( 2.2)	14 ( 3.3)	0.827	7 ( 2.0)	9 ( 5.6)	0.063
Peripheral Vasc. Disease, *n* (%)	10 ( 2.0)	4 ( 4.4)	6 ( 1.4)	0.147	4 ( 1.2)	6 ( 3.7)	0.112
Smoking, *n* (%)	140 (27.5)	22 (24.4)	118 (28.2)	0.558	83 (23.9)	57 (35.2)	0.011
Patient outcomes							
Hospital length of stay, days (median [IQR])	7.00 [3.24, 13.00]	5.00 [2.00, 8.00]	8.00 [4.00, 14.00]	<0.001	5.00 [3.00, 8.00]	17.00 [11.00, 25.00]	<0.001
Modified Rankin Scale Score at Hospital Discharge, *n*(%)				0.093			<0.001
0 to 2: Looks after own affairs without assistance	362 (71.1)	63 (70.0)	299 (71.4)		310 (89.3)	52 (32.1)	
3: Ambulates unassisted, needs some help with own affairs	47 ( 9.2)	5 ( 5.6)	42 (10.0)		18 ( 5.2)	29 (17.9)	
4 to 5: Unable to ambulate unassisted, needs assistance with own bodily care	57 (11.2)	9 (10.0)	48 (11.5)		8 ( 2.3)	49 (30.2)	
6: dead	43 ( 8.4)	13 (14.4)	30 ( 7.2)		11 ( 3.2)	32 (19.8)	
30‐day mortality, *n* (%)	46 ( 9.1)	13 (14.4)	33 ( 7.9)	0.079	11 ( 3.2)	35 (21.7)	<0.001

### Timing of neurologic manifestations by Covid‐19 severity

Table 2 summarizes the neurologic manifestations that occurred at onset and any time during Covid‐19. Patients with severe disease had a higher frequency of neurologic manifestations (124 [92.5%] vs. 295 [78.9%], *P* = 0.001), which resulted primarily from a higher frequency of encephalopathy in severe Covid‐19 (113 (84.3%) vs. 49 (13.1%) patients, *P* < 0.001).

**Table 2:** Timing of neurologic manifestations by Covid‐19 severity.

	At time of Covid‐19 symptom onset				Any time during Covid‐19			
	Overall	Non‐severe Covid‐19 disease	Severe Covid‐19 disease	*P*	Overall	Non‐severe Covid‐19 disease	Severe Covid‐19 disease	*P*
*N*	509	375	134		509	375	134	
Any neurologic manifestations *n*(%)	215 ( 42.2)	160 ( 42.7)	55 ( 41.0)	0.822	419 ( 82.3)	295 ( 78.7)	124 ( 92.5)	0.001
Number of neurologic manifestations *n*(%)				0.595				<0.001
0	294 ( 57.8)	215 ( 57.3)	79 ( 59.0)		77 ( 15.1)	70 ( 18.7)	7 ( 5.2)	
1	145 ( 28.5)	106 ( 28.3)	39 ( 29.1)		146 ( 28.7)	116 ( 30.9)	30 ( 22.4)	
2	53 ( 10.4)	39 ( 10.4)	14 ( 10.4)		133 ( 26.1)	93 ( 24.8)	40 ( 29.9)	
3	13 ( 2.6)	12 ( 3.2)	1 ( 0.7)		101 ( 19.8)	71 ( 18.9)	30 ( 22.4)	
4 or more	4 ( 0.8)	3 ( 0.8)	1 ( 0.7)		52 ( 10.2)	25 ( 6.7)	27 ( 20.1)	
Myalgias *n* (%)	134 ( 26.4)	99 ( 26.6)	35 ( 26.1)	1	228 ( 44.8)	172 ( 45.9)	56 ( 41.8)	0.476
Headache *n* (%)	84 ( 16.5)	64 ( 17.1)	20 ( 14.9)	0.662	192 ( 37.7)	149 ( 39.7)	43 ( 32.1)	0.143
Encephalopathy *n* (%)	9 ( 1.8)	8 ( 2.1)	1 ( 0.7)	0.507	162 ( 31.8)	49 ( 13.1)	113 ( 84.3)	<0.001
Dizziness/vertigo *n* (%)	26 ( 5.1)	24 ( 6.4)	2 ( 1.5)	0.047	151 ( 29.7)	111 ( 29.6)	40 ( 29.9)	1
Dysgeusia *n* (%)	24 ( 4.7)	17 ( 4.5)	7 ( 5.2)	0.931	81 ( 15.9)	64 ( 17.1)	17 ( 12.7)	0.293
Anosmia *n* (%)	18 ( 3.5)	14 ( 3.7)	4 ( 3.0)	0.897	58 ( 11.4)	47 ( 12.5)	11 ( 8.2)	0.233
Syncope *n* (%)	6 ( 1.2)	4 ( 1.1)	2 ( 1.5)	1	22 ( 4.3)	15 ( 4.0)	7 ( 5.2)	0.726
Rhabdomyolysis *n* (%)	2 ( 0.4)	1 ( 0.3)	1 ( 0.7)	1	18 ( 3.5)	1 ( 0.3)	17 ( 12.7)	<0.001
Orthostatic hypotension *n* (%)	—	—	—	—	16 ( 3.1)	10 ( 2.7)	6 ( 4.5)	0.458
Ischemic stroke *n* (%)	—	—	—	—	7 ( 1.4)	2 ( 0.5)	5 ( 3.7)	0.022
Movement disorder *n* (%)	1 ( 0.2)	1 ( 0.3)	0 ( 0.0)	1	4 ( 0.8)	2 ( 0.5)	2 ( 1.5)	0.610
Seizure *n* (%)	2 ( 0.4)	2 ( 0.5)	0 ( 0.0)	0.966	4 ( 0.8)	4 ( 1.1)	0 ( 0.0)	0.528
Focal motor deficits *n* (%)	—	—	—	—	3 ( 0.6)	2 ( 0.5)	1 ( 0.7)	1
Ataxia *n* (%)	—	—	—	—	2 ( 0.4)	1 ( 0.3)	1 ( 0.7)	1
Polyneuropathy *n* (%)	—	—	—	—	2 ( 0.4)	0 ( 0.0)	2 ( 1.5)	0.117
Encephalitis *n* (%)	—	—	—	—	1 ( 0.2)	1 ( 0.3)	0 ( 0.0)	1
Focal sensory deficits *n* (%)	1 ( 0.2)	0 ( 0.0)	1 ( 0.7)	0.591	1 ( 0.2)	0 ( 0.0)	1 ( 0.7)	0.591
Hemorrhagic stroke *n* (%)	—	—	—	—	1 ( 0.2)	0 ( 0.0)	1 ( 0.7)	0.591
Polyradiculitis *n* (%)	1 ( 0.2)	0 ( 0.0)	1 ( 0.7)	0.591	1 ( 0.2)	0 ( 0.0)	1 ( 0.7)	0.591

Myalgias, headache, encephalopathy, dizziness, dysgeusia, and anosmia together accounted for 95.8% of all neurologic manifestations at Covid‐19 onset and 91.4% during the course of the disease. Conversely, ischemic and hemorrhagic stroke, movement disorders, focal motor and sensory deficits, ataxia, and seizures were uncommon, affecting between 0.2 and 1.4% of patients each. No cases of Guillain‐Barre syndrome or acute demyelinating encephalomyelitis (ADEM) were identified. The median number of neurologic manifestations per patient was 2 [1, 3].

### Laboratory markers in Covid‐19 patients with and without neurologic manifestations

**Figure 1:**
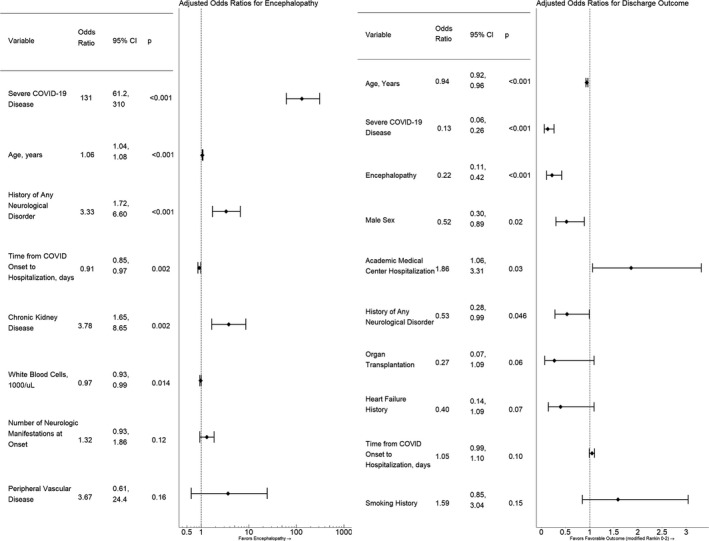
Parsimoniously Adjusted Models of Encephalopathy Occurrence and Favorable Discharge Functional Outcome (Modified Rankin Scale Score 0–2). (A) Parsimoniously adjusted model of encephalopathy occurrence. *A priori* variables included: severe COVID‐19 disease; age; history of any neurological disorder; time from COVID‐19 onset to hospitalization; serum white blood cell count; number of neurologic manifestations at COVID‐19 onset; occurrence of anosmia, dysgeusia, or headache at COVID‐19 onset; male sex; C‐reactive protein; and D‐dimer. In addition to the *a priori* variables, the following variables were univariately associated with encephalopathy at *P* ≤ 0.15 and were considered in the backward stepwise Akaike Information Criteria algorithm used to generate the parsimonious model: history of smoking, dyslipidemia, diabetes mellitus, hypertension, cerebrovascular disease, peripheral vascular disease, chronic kidney disease, heart failure, cancer, and organ transplantation; and procalcitonin level. Only those variables that satisfied the Akaike Information Criteria algorithm for parsimonious model inclusion are listed. (B) Parsimoniously adjusted model of favorable functional outcome at discharge (modified Rankin Scale Score 0 to 2). *A priori* variables included: age, severe COVID‐19 disease, occurrence of encephalopathy, male sex, history of any neurological disorder, academic medical center hospitalization, time from COVID‐19 onset to hospitalization, and race. In addition to the *a priori* variables, the following variables were univariately associated with encephalopathy at *P* ≤ 0.15 and were considered in the backward stepwise Akaike Information Criteria algorithm used to generate the parsimonious model: ethnicity; history of heart failure, organ transplantation, smoking, dyslipidemia, hypertension, cerebrovascular disease, peripheral vascular disease, coronary artery disease, chronic kidney disease, and cancer. Only those variables that satisfied the Akaike Information Criteria algorithm for parsimonious model inclusion are listed.

Table 3 summarizes serum laboratory results evaluating inflammation routinely performed in Covid‐19 patients in our system, stratified by presence of any neurologic manifestations or encephalopathy. Only patients with encephalopathy had higher white blood cell count, C‐reactive protein, D‐dimer, ferritin, and procalcitonin levels than those without encephalopathy; however, these associations did not remain after adjustment in the logistic regression model of encephalopathy (Fig. 1A).

**Table 3:** Hospital Admission Laboratory markers of inflammation in Covid‐19 patients with and without neurologic manifestations.

	Overall	No neurologic manifestation	Any neurologic manifestation	*P*	No encephalopathy	Encephalopathy	*P*
*n*	509	90	419		347	162	
White blood cell, (median [IQR]), Reference: 3.5–10.5 1000/*µ*L	6.3 [4.6, 8.3]	6.3 [4.4, 9.4]	6.3 [4.7, 8.2]	0.863	5.9 [4.6, 7.7]	6.9 [5.0, 9.4]	0.001
Platelet, (median [IQR]), Reference: 140–390 1000/*µ*L	199 [162, 249]	206 [170, 248]	198 [162, 249]	0.542	200 [165, 247]	199 [159, 255]	0.68
C‐Reactive Protein, (median [IQR]), Reference: 0.0–0.5 mg/dL	10.45 [3.73, 29.68]	10.15 [3.58, 44.89]	10.60 [4.00, 28.38]	0.948	9.40 [3.30, 29.64]	13.80 [4.60, 29.80]	0.033
D‐dimer, (median [IQR]), Reference: 0–230 ng/mL	296 [185, 583]	332 [192, 595]	285 [184, 570]	0.568	265 [166, 448]	436 [240, 847]	<0.001
Ferritin, (median [IQR]), Reference: 24–336 ng/mL	448 [196, 949]	398 [159, 738]	468 [205, 976]	0.153	352 [187, 816]	633 [257, 1244]	0.002
Procalcitonin, (median [IQR]), Reference: ≤0.065 ng/mL	0.10 [0.02, 0.24]	0.10 [0.02, 0.39]	0.10 [0.02, 0.24]	0.757	0.07 [0.02, 0.18]	0.17 [0.08, 0.41]	<0.001

### Parsimoniously adjusted models of neurologic manifestations

In a parsimoniously adjusted model, younger patients (adjusted OR 0.982; 95% CI 0.968, 0.996 per year; *P* = 0.014) and those with severe Covid‐19 disease (adjusted OR 4.02; 95% CI 2.04, 8.89; *P* < 0.001) were more likely to develop any neurologic manifestations (Table S2). In contrast, older patients were more likely to develop encephalopathy (adjusted OR 1.06; 95% CI 1.04, 1.08 per year; *P* < 0.001). The parsimoniously adjusted model of encephalopathy also suggested that severe Covid‐19 disease (adjusted OR 131; 95% CI 61.2, 310; *P* < 0.001), a history of any neurological disorder or chronic kidney disease, lower admission serum white blood cell count, and shorter time from Covid‐19 onset to hospitalization were associated with greater likelihood of encephalopathy (Fig. 1A). Due in part to logistical challenges and restrictions on diagnostic testing due to pandemic infection prevention measures, the specific pathophysiology of the neurologic manifestations was not established with certainty in the majority of cases. For instance, in‐person neurology or neurosurgical consultation was obtained only in 30 (5.9%), brain CT in 46 (9.0%), brain MRI in 16 (3.1%), electroencephalograms in 6 (1.2%), and lumbar puncture in 2 (0.4%) of the 509 patients.

### Differences between the Academic Medical Center (AMC) and other hospitals of the network

Table 4 summarizes the differences between 254 (49.9 %) patients treated at our AMC versus the other nine hospitals in our system. Patients at the AMC were younger but had greater comorbidities and were more often black or African American, consistent with our regional demographics. Patients at the AMC more often had neurologic manifestations at the time of hospitalization and had an overall higher frequency of neurologic manifestations during the course of Covid‐19; however, there was no significant difference in the occurrence of encephalopathy. Chronic medications prior to hospitalization and experimental treatments provided during hospitalization are listed in Table S3. Rates of mechanical ventilation were similar between the AMC and the other hospitals; however, do not resuscitate, do not intubate, and comfort measures only orders were less frequent at the AMC. Mortality 30 days after hospitalization was lower at the AMC (5.1% vs. 12.9%, *P* = 0.003) and functional outcome at discharge was more favorable at the AMC.

**Table 4:** Covid‐19 patient characteristics comparing academic medical center versus other hospitals.

	Overall	Academic medical center	All other hospitals	*P*
*n*	509	254	255	
Age, years (mean (SD))	58.51 (16.93)	57.00 (16.39)	59.98 (17.34)	0.047
Male sex, *n* (%)	281 (55.2)	138 (54.3)	143 (56.1)	0.759
Race, *n* (%)				<0.001
White	268 (52.7)	78 (30.7)	190 (74.5)	
Black or African American	151 (29.7)	116 (45.7)	35 (13.7)	
Asian	18 ( 3.5)	12 ( 4.7)	6 ( 2.4)	
Native Hawaiian or Other Pacific Islander	1 ( 0.2)	0 ( 0.0)	1 ( 0.4)	
Other	53 (10.4)	35 (13.8)	18 ( 7.1)	
Unknown	1 ( 0.2)	1 ( 0.4)	0 ( 0.0)	
Ethnicity, *n* (%)				0.793
Not Hispanic or Latino	384 (75.4)	193 (76.0)	191 (74.9)	
Hispanic or Latino	107 (21.0)	51 (20.1)	56 (22.0)	
Declined to Provide	18 ( 3.5)	10 ( 3.9)	8 ( 3.1)	
Time from COVID Onset to Hospitalization, days (median [IQR])	7.00 [4.00, 9.59]	7.00 [4.00, 10.00]	6.00 [3.00, 9.00]	0.012
Neurologic manifestations				
Any neurologic manifestations at symptom onset, *n* (%)	215 (42.2)	117 (46.1)	98 (38.4)	0.098
Number of neurologic manifestations at symptom onset, (median [IQR])	0 [0, 1]	0 [0, 1]	0 [0, 1]	0.188
Any neurologic manifestations at time of hospitalization, *n* (%)	319 (62.7)	180 (70.9)	139 (54.5)	<0.001
Any neurologic manifestations during Covid‐19, *n* (%)	419 (82.3)	222 (87.4)	197 (77.3)	0.004
Number of neurologic manifestations during Covid‐19, (median [IQR])	2 [1, 3]	2 [1, 3]	1 [1, 2]	<0.001
Encephalopathy, *n* (%)	162 (31.8)	86 (33.9)	76 (29.8)	0.375
Comorbidities prior to admission				
Number of medical comorbidities, (median [IQR])	6.00 [3.00, 9.00]	6.50 [4.00, 10.00]	5.00 [3.00, 8.00]	<0.001
History of any neurological disorder, *n* (%)	134 (26.3)	67 (26.4)	67 (26.3)	1
Obesity (body‐mass index >30), *n* (%)	262 (51.5)	127 (50.0)	135 (52.9)	0.565
Hospital treatment				
Mechanical ventilation, *n* (%)	134 (26.3)	72 (28.3)	62 (24.3)	0.351
Extracorporeal membrane oxygenation, *n* (%)	5 ( 1.0)	5 ( 2.0)	0 ( 0.0)	0.072
Renal replacement therapy initiated, *n* (%)	27 ( 5.3)	18 ( 7.1)	9 ( 3.5)	0.111
Do not resuscitate order, *n* (%)	73 (14.4)	27 (10.6)	46 (18.0)	0.024
Do no intubate order, *n* (%)	38 ( 7.5)	11 ( 4.3)	27 (10.6)	0.012
Transition to comfort measures only, *n* (%)	30 ( 5.9)	9 ( 3.6)	21 ( 8.2)	0.039
Patient outcomes				
Hospital Length of Stay, days (median [IQR])	7.00 [3.24, 13.00]	7.00 [4.00, 14.79]	7.00 [3.00, 12.00]	0.106
Modified Rankin Scale Score at Hospital Discharge, *n* (%)				0.009
0 to 2: Looks after own affairs without assistance	362 (71.1)	189 (74.4)	173 (67.8)	
3: Ambulates unassisted, needs some help with own affairs	47 ( 9.2)	28 (11.0)	19 ( 7.5)	
4 to 5: Unable to ambulate unassisted, needs assistance with own bodily care	57 (11.2)	25 ( 9.8)	32 (12.5)	
6: dead	43 ( 8.4)	12 ( 4.7)	31 (12.2)	
30‐day mortality, *n* (%)	46 ( 9.1)	13 ( 5.1)	33 (12.9)	0.003

### Patient outcomes

Patients with neurologic manifestations experienced longer hospitalization (8 [4, 14] vs. 5 [2, 8] days, *P* < 0.001) and, in particular, those with encephalopathy had median hospitalizations over threefold longer (Table 1). Discharge functional outcome and mortality was not significantly different between those with and without any neurologic manifestations. No single neurologic manifestation, besides encephalopathy, was significantly associated with worse discharge functional outcome or greater mortality. However, in the parsimoniously adjusted model of discharge functional outcome (Fig. 1B), patients with encephalopathy were independently less likely to experience favorable functional outcome (mRS 0–2; adjusted OR 0.22, 95% CI 0.11–0.42; *P* < 0.001). Other factors associated with favorable functional outcome at discharge, after adjustment were: absence of severe Covid‐19 disease, younger age, female sex, absence of pre‐existing neurologic disorders, and being hospitalized at the AMC. In a model of mortality adjusting for severe Covid‐19 disease, age, and hospitalization at the AMC, the occurrence of encephalopathy remained independently associated with greater likelihood of death at 30 days after hospitalization (adjusted OR 2.92, 95% CI 1.17, 7.57; *P* = 0.02).

## Discussion

This study highlights the high frequency and range of neurologic manifestations, which occurred in more than four fifths of Covid‐19 patients hospitalized in our hospital network system. These results expand findings of neurologic manifestations in 36.4% of hospitalized Covid‐19 patients in China and 57.4% in Europe^16^, ^17^, albeit with increased prevalence in our US cohort. Differences in frequencies may be caused by genetic factors including polymorphism in expression of the viral receptor angiotensin‐converting enzyme 2 (ACE 2) in the nervous system, and possibly, SARS‐CoV‐2 strain variations.^18^ In addition, our hospital network system was never stressed beyond capacity due to surge preparation and most patients had moderate disease, with only one quarter developing severe respiratory distress requiring mechanical ventilation.^19^ This may have allowed for more detailed evaluation and identification of neurologic manifestations.

The fact that any neurologic manifestations as a whole were more likely to occur in younger people is surprising, and could potentially be explained by greater clinical emphasis on the risk of respiratory failure than other symptoms in older patients. Alternatively, early neurologic manifestations such as myalgia, headache, or dizziness may have prompted earlier medical care. In contrast, encephalopathy was more frequent in older patients. Risk factors for encephalopathy also included severe Covid‐19 disease and history of any neurological disorder or chronic kidney disease. This is consistent with recent literature identifying higher rates of mortality in Covid‐19 patients with pre‐existing chronic neurological disorders.^20^

The increased morbidity and mortality associated with encephalopathy, independent of respiratory severity, parallels previous literature in sepsis‐associated encephalopathy and delirium‐associated mortality^11^, ^21^ and emphasizes its relevance in Covid‐19. We also found that encephalopathy in Covid‐19 was associated with triple the hospital length of stay. Broad recognition and screening for encephalopathy as a contributor to disease severity in Covid‐19 may have utility in resource allocation and potential to improve patient outcomes. Furthermore, our findings emphasize the broader need to develop interventions that target encephalopathy as a component of multi‐organ system medical illness.

The cause of encephalopathy could not be determined with certainty given the lack of extensive diagnostic neurologic testing for most patients in this study due to ongoing pandemic restrictions. However, the most likely etiology of encephalopathy in patients with Covid‐19 is multifactorial, including systemic disease and inflammation, coagulopathy, direct neuroinvasion by the virus, endotheliitis and possibly post‐infectious auto‐immune mechanisms.^5^ Additionally, traditional risk factors associated with intensive care unit delirium and encephalopathy also need to be taken into account, including sedation and analgesia doses, disruption of sleep/wake cycles, and infectious complications.^22^, ^23^ Critical illness encephalopathy, in general, can result from multiple mechanisms or combinations of mechanisms that remain an unresolved area of basic science research.^24^

Our hospital network system includes an AMC and nine other hospitals. There was no meaningful difference in severity of disease between the AMC and the other hospitals, but patients at the AMC had better functional outcomes and lower 30‐day mortality. One potential explanation for this finding may be related to specialty care, access to resources, and lower rates of do not resuscitate/intubate and comfort measures‐only orders. Our AMC is also the largest hospital within the NMHC system with the greatest number of ICU beds, which may confer a survival benefit to critically ill patients with Covid‐19.^25^ A similar difference has been observed in patients with sepsis, who had better outcomes and lower mortality when treated by tertiary versus non‐tertiary hospitals.^26^ Early adoption and implementation of uniform treatment protocols across hospital networks driven by AMCs may be a means to improve outcome and lower mortality of Covid‐19 patients that deserves further investigations.

Our study has limitations, including its retrospective nature, and the fact that fewer than 6% of patients were evaluated by neurologists or neurosurgeons. Since most patients were admitted to dedicated Covid‐19 wards or ICUs with strict infection control precautions in place, access to brain CT or MRI was not as readily available as for other patients with neurologic diseases. This limited a more complete neurologic work up in many Covid‐19 patients. Additionally, patients were cared for at 10 different hospitals and there may have been varying rates of local geographic infection severity. However, this provided us with a more generalized view of the neurologic manifestations in Covid‐19 patients and could identify opportunities for regionalized resource allocation and preparedness protocols in a large hospital network system.

Only 9 months into the pandemic, the long‐term effects of Covid‐19 on the nervous system remain uncertain. Our results suggest that, of all neurologic manifestations, encephalopathy is associated with a worse functional outcome in hospitalized patients with Covid‐19, and may have lasting effects.^12^ Long‐term follow‐up is necessary to assess the true burden of encephalopathy in these patients. Whether milder forms occur in non‐hospitalized individuals with Covid‐19 who complain of protracted inability to concentrate or decreased short term memory (referred to as ‘brain fog’) warrants further evaluation.^27^ Prospective cognitive and neurologic‐focused evaluations through specialized clinics dedicated to further diagnostic assessment and tailored rehabilitation needs could play a significant role in recovery from this pandemic.

## Conflict of Interest

The authors report no conflict of interest pertaining to this publication.

## Authors Contributions

All authors contributed to the design of the study, collection and analysis of the data and redaction of the manuscript.

## Supporting information


**Table S1.** Additional patient characteristics by presence of neurologic manifestations and encephalopathy.
**Table S2.** Details of *A Priori* and *Parsimonious* Adjusted Binary Logistic Regression Models of Any Neurologic Manifestation, Encephalopathy, and Favorable Functional Outcome at Discharge.
**Table S3.** Additional Covid‐19 Patient Characteristics Comparing Academic Medical Center vs. Other Hospitals
