# Supplementary material for: Frequent neurologic manifestations and encephalopathy‐associated morbidity in Covid‐19 patients
Source: Ann Clin Transl Neurol. 2020 Oct 5;7(11):2221–30. doi: 10.1002/acn3.51210 (PMC7664279; doi:10.1002/acn3.51210)
Supplement: Supplementary file 1 — Table S1. Additional patient characteristics by presence of neurologic manifestations and encephalopathy. Table S2. Details of A Priori and Parsimonious Adjusted Binary Logistic Regression Models of Any Neurologic Manifestation, Encephalopathy, and Favorable Functional Outcome at Discharge. Table S3. Additional Covid‐19 Patient Characteristics Comparing Academic Medical Center vs. Other Hospitals [file ACN3-7-2221-s001.doc]

**Supplemental Table 1. Additional Patient Characteristics by presence of neurologic manifestations and encephalopathy.**

|  | **Overall** | **No Neurologic Manifestation** | **Any Neurologic Manifestation** | **p** | **No Encephalopathy** | **Encephalopathy** | **p** |
| --- | --- | --- | --- | --- | --- | --- | --- |
| n | 509 | 90 | 419 |  | 347 | 162 |  |
| **Additional Medical Comorbidities** | | | | | | | |
| Alcohol or Substance Abuse, n (%) | 17 ( 3.3) | 2 ( 2.2) | 15 ( 3.6) | 0.744 | 13 ( 3.7) | 4 ( 2.5) | 0.63 |
| Autoimmune disease, n (%) | 29 ( 5.7) | 4 ( 4.4) | 25 ( 6.0) | 0.753 | 23 ( 6.6) | 6 ( 3.7) | 0.262 |
| COPD or Emphysema, n (%) | 75 (14.7) | 14 (15.6) | 61 (14.6) | 0.938 | 51 (14.7) | 24 (14.8) | 1 |
| Coronary Artery Disease, n (%) | 56 (11.0) | 8 ( 8.9) | 48 (11.5) | 0.603 | 34 ( 9.8) | 22 (13.6) | 0.264 |
| HIV, n (%) | 6 ( 1.2) | 0 ( 0.0) | 6 ( 1.4) | 0.546 | 5 ( 1.4) | 1 ( 0.6) | 0.718 |
| Interstitial lung disease or Other Lung Disease, n (%) | 9 ( 1.8) | 2 ( 2.2) | 7 ( 1.7) | 1 | 6 ( 1.7) | 3 ( 1.9) | 1 |
| Obesity (body-mass index > 30), n (%) | 262 (51.5) | 39 (43.3) | 223 (53.2) | 0.113 | 179 (51.6) | 83 (51.2) | 1 |
| **Hospital Treatments** | | | | | | | |
| Hydroxychloroquine, n (%) | 265 (52.1) | 52 (57.8) | 213 (50.8) | 0.28 | 174 (50.1) | 91 (56.2) | 0.241 |
| Remdesivir, n (%) | 21 ( 4.1) | 5 ( 5.6) | 16 ( 3.8) | 0.646 | 11 ( 3.2) | 10 ( 6.2) | 0.178 |
| Sarilumab, n (%) | 8 ( 1.6) | 2 ( 2.2) | 6 ( 1.4) | 0.936 | 8 ( 2.3) | 0 ( 0.0) | 0.117 |
| Tocilizumab, n (%) | 37 ( 7.3) | 5 ( 5.6) | 32 ( 7.6) | 0.641 | 21 ( 6.1) | 16 ( 9.9) | 0.172 |

**Supplemental Table 2: Details of *A Priori* and *Parsimonious* Adjusted Binary Logistic Regression Models of Any Neurologic Manifestation, Encephalopathy, and Favorable Functional Outcome at Discharge**.

| **Any Neurologic Manifestation^1^** | | | | | | |
| --- | --- | --- | --- | --- | --- | --- |
|  | **A Priori Model** | | | **Parsimonious Model** | | |
| Variable | Odds Ratio | 95% Confidence Interval | p | Odds Ratio | 95% Confidence Interval | p |
| Severe Covid-19 Disease | 4.11 | 202, 9.29 | <0.001 | 4.02 | 2.04, 8.89 | <0.001 |
| Age, years | 0.97 | 0.96, 0.99 | 0.002 | 0.982 | 0.968, 0.996 | 0.014 |
| White Blood Cell Count (1000/µL) | 0.98 | 0.94, 1.003 | 0.16 | 0.978 | 0.934, 1.00 | 0.13 |
| C-Reactive Protein (mg/dL) | 1.00 | 0.99, 1.002 | 0.25 | -- | -- | -- |
| Male Sex | 0.78 | 0.45, 1.34 | 0.37 | -- | -- | -- |
| D-Dimer (ng/mL) | 1.00 | 1.00, 1.00 | 0.82 | -- | -- | -- |
| History of Any Neurological Disorder | 0.99 | 0.56, 1.80 | 0.97 | -- | -- | -- |
| Hispanic Ethnicity | **--** | **--** | **--** | 1.69 | 0.87, 3.58 | 0.14 |
|  | | | | | | |
| **Encephalopathy^2^** | | | | | | |
|  | **A Priori Model** | | | **Parsimonious Model** | | |
| Variable | Odds Ratio | 95% Confidence Interval | p | Odds Ratio | 95% Confidence Interval | p |
| Severe Covid-19 Disease | 109 | 49.6, 266 | <0.001 | 131 | 61.2, 310 | <0.001 |
| Age, years | 1.06 | 1.04, 1.08 | <0.001 | 1.06 | 1.04, 1.08 | <0.001 |
| History of Any Neurological Disorder, n(%) | 2.91 | 1.44, 6.01 | 0.003 | 3.33 | 1.72, 6.60 | <0.001 |
| Time from COVID Onset to Hospitalization, days | 0.91 | 0.85, 0.97 | 0.004 | 0.91 | 0.85, 0.97 | 0.002 |
| White Blood Cell Count (1000/µL) | 0.98 | 0.94, 1.00 | 0.08 | 0.97 | 0.93, 0.99 | 0.014 |
| Number of Neurologic Manifestations at Onset | 1.51 | 0.87, 2.57 | 0.14 | 1.32 | 0.93, 1.86 | 0.12 |
| Anosmia, Dysguesia, or Headache at Covid-19 Onset | 0.61 | 0.19, 1.95 | 0.40 | -- | -- | -- |
| Male Sex | 1.18 | 0.63, 2.21 | 0.61 | -- | -- | -- |
| C-Reactive Protein (mg/dL) | 1.00 | 0.99, 1.01 | 0.74 | -- | -- | -- |
| D-Dimer (ng/mL) | 1.00 | 1.00, 1.00 | 0.87 | -- | -- | -- |
| Chronic Kidney Disease | -- | -- | -- | 3.78 | 1.65, 8.65 | 0.002 |
| Peripheral Vascular Disease | -- | -- | -- | 3.67 | 0.61, 24.4 | 0.16 |
|  | | | | | | |
| **Favorable Functional Outcome at Discharge (modified Rankin Scale Score 0 to 2)^3^** | | | | | | |
|  | **A Priori Model** | | | **Parsimonious Model** | | |
| Variable | Odds Ratio | 95% Confidence Interval | p | Odds Ratio | 95% Confidence Interval | p |
| Age, years | 0.94 | 0.92, 0.96 | <0.001 | 0.94 | 0.92, 0.96 | <0.001 |
| Severe Covid-19 Disease | 0.13 | 0.06, 0.28 | <0.001 | 0.13 | 0.06, 0.27 | <0.001 |
| Encephalopathy | 0.21 | 0.11, 0.41 | <0.001 | 0.22 | 0.11, 0.42 | <0.001 |
| Male Sex | 0.54 | 0.31, 0.93 | 0.03 | 0.53 | 0.30, 0.92 | 0.02 |
| History of Any Neurological Disorder, n(%) | 0.56 | 0.30, 1.04 | 0.07 | 0.54 | 0.29, 0.998 | 0.049 |
| Hospitalized at the Academic Medical Center | 1.74 | 0.94, 3.26 | 0.08 | 2.05 | 1.18, 3.65 | 0.01 |
| Time from COVID Onset to Hospitalization, days | 1.04 | 0.99, 1.10 | 0.12 | -- | -- | -- |
| Race  White  Black  Other  Unknown | 1.07  Reference  1.68  >1000 | 0.53, 2.14  --  0.70, 4.12  <0.001, >10000 | 0.84  --  0.25  0.99 | -- | -- | -- |
| Heart Failure History | -- | -- | -- | 0.38 | 0.14, 1.01 | 0.054 |
| Organ Transplantation | -- | -- | -- | 0.27 | 0.07, 1.05 | 0.06 |
| Smoking History | -- | -- | -- | 1.58 | 0.85, 3.02 | 0.16 |

For each model, *a priori* variables included: severe Covid-19 disease, age, male sex, and history of any neurological disorder.

1. For the model of any neurologic manifestations, additional *a priori* variables included: white blood cell count, D-dimer, and C-reactive protein. In addition to the a priori variables, the following variables were univariately associated with having a neurologic manifestation at p≤0.15 and were considered in the backward stepwise algorithm used to generate the parsimonious model: ethnicity, obesity, cerebrovascular disease, peripheral vascular disease, and time from COVID onset to hospitalization.

2. For the model of encephalopathy, additional *a priori* variables included: white blood cell count; D-dimer; C-reactive protein; time from Covid-19 onset to hospitalization; number of neurologic manifestations at Covid-19 onset; and occurrence of anosmia, dysgeusia, or headache at Covid-19 onset. In addition to the a priori variables, the following variables were univariately associated with encephalopathy at p≤0.15 and were considered in the backward stepwise algorithm used to generate the parsimonious model: history of smoking, dyslipidemia, diabetes mellitus, hypertension, cerebrovascular disease, peripheral vascular disease, chronic kidney disease, heart failure, cancer, and organ transplantation; and procalcitonin level.

3. For the model of discharge functional outcome, additional *a priori* variables included: occurrence of encephalopathy, race, hospitalization at the academic medical center, and time from Covid-19 onset to hospitalization. In addition to the a priori variables, the following variables were univariately associated with favorable functional outcome at discharge at p≤0.15 and were considered in the backward stepwise algorithm used to generate the parsimonious model: ethnicity and history of smoking, dyslipidemia, hypertension, cerebrovascular disease, peripheral vascular disease, coronary artery disease, chronic kidney disease, heart failure, cancer, and organ transplantation.

**Supplemental Table 3: Additional Covid-19 Patient Characteristics Comparing Academic Medical Center vs. Other Hospitals**

|  | **Overall** | **Academic Medical Center** | **All Other Hospitals** | **p** |
| --- | --- | --- | --- | --- |
| n | 509 | 254 | 255 |  |
| **Chronic Medications Prior to Admission** | | | | |
| ACE inhibitor, n (%) | 68 (13.4) | 37 (14.6) | 31 (12.2) | 0.504 |
| Angiotensin II Receptor Blocker, n (%) | 51 (10.0) | 35 (13.8) | 16 ( 6.3) | 0.008 |
| Corticosteroids, n (%) | 43 ( 8.4) | 30 (11.8) | 13 ( 5.1) | 0.01 |
| Immunosuppressant, n (%) | 39 ( 7.7) | 32 (12.6) | 7 ( 2.7) | <0.001 |
| **Additional Hospital Treatments** | | | | |
| Hydroxychloroquine, n (%) | 265 (52.1) | 138 (54.3) | 127 (49.8) | 0.351 |
| Remdesivir, n (%) | 21 ( 4.1) | 10 ( 3.9) | 11 ( 4.3) | 1 |
| Sarilumab, n (%) | 8 ( 1.6) | 2 ( 0.8) | 6 ( 2.4) | 0.288 |
| Tocilizumab, n (%) | 37 ( 7.3) | 25 ( 9.8) | 12 ( 4.7) | 0.039 |
